# Supplementary material for: The Implementation of Antimicrobial Consumption Surveillance and Stewardship in Human Healthcare in Post-Soviet States: A Systematic Review
Source: Antibiotics (Basel). 2025 Jul 25;14(8):749. doi: 10.3390/antibiotics14080749 (PMC12382636; doi:10.3390/antibiotics14080749)
Supplement: Supplementary file 1 [file antibiotics-14-00749-s001.zip › Supplementary material-TableS2 .docx]

**Supplementary file**

Table S2. Categorization of AMC and AMS in available NAPs

| **Country** | **Extract for the NAP on AMC*** | **Categorization**** | **Extract for the NAP on AMS*** | **Categorization**** |
| --- | --- | --- | --- | --- |
| Armenia [50] | **Strategic direction: Monitoring the use of antimicrobials among humans and animals and improving control of their circulation.** Expected results in the NAP: Establishing the foundations of a system for monitoring the use of antimicrobials in animal health and agriculture. Availability of reliable data on the use of antimicrobials among the population and creating the basis for a system to monitor the use of antimicrobials among animals. | Strategic objective | **Strategic Direction 7. Development of mechanisms for implementing an Antimicrobial Stewardship system.** Strategy activities: Development and implementation of clinical guidelines (standards, patient management protocols) for various diseases. Development of legal foundations and appropriate procedures for mandatory antimicrobial stewardship in healthcare organizations to ensure: Selection of the correct drug Correct dosage Correct route of administration Correct duration of treatment Reduction in the use of broad-spectrum antimicrobials Analysis of prescriptions and discussion with physicians | Strategic objective |
| Georgia [51] | **Task III: Surveillance of antimicrobial consumption and improvement of rational use practices.** Strengthening surveillance of antimicrobial (antibiotic) consumption, routine analysis of antibiotic usage data, and supporting the ban on over-the-counter antibiotic sales without prescription, along with comprehensive mechanisms and regulations for its enforcement, can be considered one of the keys to successful policy. Based on this, surveillance of the drug dispensing process, feedback to clinicians, collection, monitoring, and analysis of prescription data will be implemented, allowing for targeted interventions to optimize the rational use of antimicrobials. Antibiotics will be prescribed only when it contributes to the recovery of a specific patient. To this end, a national list of antimicrobials will be developed and implemented in accordance with the WHO Essential Medicines List and the AWaRe classification. National recommendations (guidelines) and clinical practice protocols for infectious diseases will be updated/developed. It is also planned to implement a behavioral science approach to promote proper prescription, use, and sale of antimicrobials by prescription. | Strategic objective | **Task III: Surveillance of antimicrobial consumption and improvement of rational use practices.** Strengthening surveillance of antimicrobial (antibiotic) consumption, routine analysis of antibiotic usage data, and supporting the ban on over-the-counter antibiotic sales without prescription, along with comprehensive mechanisms and regulations for its enforcement, can be considered one of the keys to successful policy. Based on this, surveillance of the drug dispensing process, feedback to clinicians, collection, monitoring, and analysis of prescription data will be implemented, allowing for targeted interventions to optimize the rational use of antimicrobials. Antibiotics will be prescribed only when it contributes to the recovery of a specific patient. To this end, a national list of antimicrobials will be developed and implemented in accordance with the WHO Essential Medicines List and the AWaRe classification. National recommendations (guidelines) and clinical practice protocols for infectious diseases will be updated/developed. It is also planned to implement a behavioral science approach to promote proper prescription, use, and sale of antimicrobials by prescription. | Strategic objective |
| Kazakhstan [52] | **Strategic direction 5: Optimization of antimicrobial drug use in human and animal health.**  There are activities in the NAP: 5.4 Analysis of antimicrobial consumption using the ATC/DDD methodology at the population level in healthcare and agriculture, as well as in healthcare organizations. Establishment of a national network of healthcare organizations for the use and consumption of antimicrobials. 5.5 Establishment of a national network of healthcare organizations for the use and consumption of antimicrobials | Strategic objective | **Strategic direction 5: Optimization of antimicrobial drug use in human and animal health.**  Tasks within the directions: Rational use of antimicrobial agents in the treatment of infectious diseases, taking into account national clinical treatment protocols, international recommendations, and results of studies on the resistance of infectious disease pathogens to antimicrobial agents in the healthcare sector. Creation of a national network of healthcare organizations for the use and consumption of antimicrobial agents. | Strategic objective |
| Kyrgyzstan [53] | **Direction (strategic): 4.3.4 Improve the epidemiological surveillance system for antimicrobial consumption:** Optimize the pharmacoepidemiological surveillance system for antimicrobial consumption; Create and improve a unified (intersectoral) country database for antimicrobial consumption in healthcare and agriculture. There are activities in the NAP plan: 4.1. Optimize the pharmacoepidemiological surveillance system for antimicrobial consumption: 4.1.1 Implement an "electronic prescription" program for antimicrobials in primary healthcare physicians' prescribing practices to ensure control over rational use of antimicrobials; 4.1.2. Optimize the surveillance system for antimicrobial consumption at outpatient and inpatient levels. 4.2 Create and improve a unified (intersectoral) country database on antimicrobial consumption in healthcare and agriculture: 4.2.1 Create sectoral databases on antimicrobial consumption; 4.2.2. Conduct studies on the prescription and use of antimicrobials in healthcare, agriculture, veterinary, and agro-industrial sectors; 4.2.3. Collect, analyze, and interpret the gathered data. | Strategic objective | **Strategic direction: Optimize the use of antimicrobial drugs and ensure access of the Kyrgyz Republic population to quality, effective, and safe antimicrobial drugs.** Activities under the strategy: Strengthen requirements for evaluating the effectiveness, safety, and quality of antimicrobial drugs. Ensure accessibility of antimicrobial drugs. Develop, disseminate, and implement guidelines for the rational use of antimicrobial drugs in healthcare. Strengthen control over prescription-based dispensing of veterinary antimicrobial drugs. Create practical guidelines for the rational use of antimicrobial drugs in animal husbandry. | Strategic objective |
| Latvia [23] | **Direction: improvement of distribution, consumption, availability and monitoring/recording of antimicrobial agents, promotion of responsible and cautious use of antibiotics.** NAP has events: To evaluate the possibilities of improving AB supply and consumption monitoring in inpatient treatment facilities, defined outcomes/action result and indicators to measure. | Strategic objective | **Direction: Improvement of distribution, consumption, accessibility, surveillance/accounting of antimicrobial agents, promotion of responsible and cautious use of antimicrobial agents.** Action plan under the strategic direction: Promote the use of EARS-Net, ESAC-Net, and ESVAC data to improve drug availability and decision-making in including antibiotics in the compensation system for outpatient treatment drugs. Review: Provide diagnoses for antibiotics that are appropriate for justified antibiotic treatment. Implement EU and national recommendations for antibiotic use. Promote the use of available information on guaranteed antibiotic consumption to analyze trends in antibiotic consumption, prescription patterns, and prescribing habits, with the aim of implementing measures in outpatient, responsible, and hospital practices to promote cautious antibiotic consumption. Improve medical personnel's understanding of responsible and cautious antibiotic consumption. | Strategic objective |
| Lithuania [24] | **Task: 17.2. Expand and improve systems for monitoring AMR, consumption of antimicrobial drugs, and healthcare-associated infections.** | Strategic objective | **Strategic objectives: 17.3. Improve the use of antimicrobial drugs in medicine through the implementation of evidence-based measures and best practice examples.** | Strategic objective |
| Moldova [54] | **General objective: 3. Strengthening mechanisms for market introduction, prescription, and dispensing of antimicrobial agents in the human, veterinary, and agricultural sectors in accordance with national and international standards for the prevention and control of antimicrobial resistance by 2027.** There are sub-tasks/Specific objectives: Specific objective 3.1. Strengthening authorization procedures for market placement and monitoring of antimicrobial consumption for human and veterinary use, as well as procedures for centralized procurement of antimicrobials for human and veterinary use by 2027; Specific objective 3.2. Monitoring the market circulation of antimicrobial agents intended for consumption in human, veterinary, and agricultural fields by 2027; Specific objective 3.3. Development and promotion of programs for monitoring antimicrobial consumption, including at the level of medical institutions, for rational prescription of antimicrobials by 2027. The Plan below includes activities for each specific objective. | Strategic objective | **General Objective 3. Strengthening mechanisms for market introduction, prescription, and dispensing of antimicrobial agents in the human, veterinary, and agricultural sectors in accordance with national and international standards for the prevention and control of antimicrobial resistance by 2027.** Specific Objective 3.1. Strengthening authorization procedures for market placement and monitoring of antimicrobial consumption for human and veterinary use, as well as procedures for centralized procurement of antimicrobials for human and veterinary use by 2027. Specific Objective 3.2. Monitoring the market circulation of antimicrobial agents intended for consumption in human, veterinary, and agricultural fields, and the circulation of resistant microorganisms in humans, animals, and food products of animal origin and feed, especially those common to humans, by 2027. Specific Objective 3.3. Development and promotion of programs for monitoring antimicrobial consumption, including at the level of medical institutions, for rational prescription of antimicrobials by 2027. | Strategic objective |
| Russian Federation [55] | **Task: VII. Improving measures to control the circulation of antimicrobial drugs, chemical and biological agents**. There is an activity within: Implementation of a traceability system for antimicrobial drugs circulation using a monitoring system for the movement of medicinal products for medical use | Strategic objective | **Task: VII. Improving measures to control the circulation of antimicrobial drugs, chemical and biological agents**. Activity within the task: Development and updating of clinical guidelines for providing medical care for infectious and parasitic diseases, taking into account the application of optimal antimicrobial therapy regimens. | Strategic objective |
| Tajikistan [56] | **Strategic objective: 4: Set up a national surveillance/monitoring centre for antibiotic consumption.** Strategic intervention: 4.1 Strengthen a national structure for obtaining an overview of the use of antibiotics within the country.  activities: 4.1.1. Write and approve terms of reference for a national coordinating group for antibiotic consumption surveillance, including collecting, aggregating and sharing data.  Strategic intervention: 4.2  Investigate and monitor antibioti**c u**se in larger hospitals and inform the pharmaceutical surveillance department of the State service for pharmaceutical activity surveillance  activities: 4.2.1.Perform recurring Point Prevalence Studies on in-patients, as the patients may receive antibiotics from various sources. | Strategic objective | **Objective 12. Improve and measure appropriate use of antimicrobial agents in health care.** 12.1. Create formal antimicrobial stewardship programmes in health care facilities. Milestone: Antimicrobial stewardship pro- grammes established in 50% of acute care facilities by Aug 2018. 12.1.1. Write generic terms of reference for antimicrobial stewardship multidisciplinary committees and teams. 12.1.2. Ensure the establishment of such com- mittees in each larger hospital including sur- rounding Primary Health Care participation. 12.1.3. Provide antimicrobial stewardship training to healthcare staff. 12.2. Improve rational use of antibiotics through modernized National Standard Treat- ment Guidelines (STGs). 12.2.1. Renew the existing system of data- base. 12.2.2. Produce Standard Treatment Guide- lines for common infections/common causes of antibiotic use. | Strategic objective |
| Turkmenistan [57] | **NAP has objectives: 2 Introduce measures to ensure rational use of antimicrobials in public health and  veterinary service; 3. Estimate quantities of antimicrobial consumption.** NAP has activities of Implementation: 5.3. Monitoring Accessibility of Antimicrobials; 5.4. Rational Use of Antimicrobials and Strengthening Surveillance of their Use. NAP also has Plan of Actions, where each activity is subdivided to several activities with timeframe and responsible org NAP has also indicators for each area in a separate table | Strategic objective | **NAP has objectives: 2 Introduce measures to ensure rational use of antimicrobials in public health and  veterinary service; 3. Estimate quantities of antimicrobial consumption.** NAP has **activities** of Implementation: 5.3. Monitoring Accessibility of Antimicrobials; 5.4. Rational Use of Antimicrobials and Strengthening Surveillance of their Use. NAP also has Plan of Actions, where each activity is subdivided to several activities with timeframe and responsible org NAP has also indicators for each area in a separate table | Strategic objective |
| Ukraine [58] | **Strategic goal 3. Implementation of a surveillance system for pathogens with antimicrobial resistance and consumption of antimicrobial drugs.** There are tasks to achieve goal 3: Improving legislation in the field regulating the procurement and prescription of antimicrobial drugs by healthcare institutions; Increasing the efficiency of the procurement system for antimicrobial drugs by healthcare institutions providing inpatient medical care; Implementation of an electronic healthcare system for collecting data on the dispensing of antimicrobial drugs ..." | Strategic objective | **Strategic goal 3. Implementation of a surveillance system for pathogens with antimicrobial resistance and consumption of antimicrobial drugs.** There are tasks to achieve goal 3: Improving legislation in the field regulating the procurement and prescription of antimicrobial drugs by healthcare institutions; Increasing the efficiency of the procurement system for antimicrobial drugs by healthcare institutions providing inpatient medical care; Implementation of an electronic healthcare system for collecting data on the dispensing of antimicrobial drugs ..." | Strategic objective |

*An online translator was used to translate extracted text from NAPs related to AMC and AMS

** Categorization was divided by reviewers to no prioritization, activity, and strategic objective
