# Supplementary material for: The Implementation of Antimicrobial Consumption Surveillance and Stewardship in Human Healthcare in Post-Soviet States: A Systematic Review
Source: Antibiotics (Basel). 2025 Jul 25;14(8):749. doi: 10.3390/antibiotics14080749 (PMC12382636; doi:10.3390/antibiotics14080749)
Supplement: Supplementary file 1 [file antibiotics-14-00749-s001.zip › Supplementary material-TableS3.docx]

**Supplementary file**

Table S3. Eligibility criteria within the PICO framework

| **PICO framework** | **Inclusion criteria** | **Exclusion criteria** |
| --- | --- | --- |
| Population | - papers on antimicrobial consumption and antimicrobial stewardship in Armenia, Azerbaijan, Belarus, Estonia, Georgia, Kazakhstan, Kyrgyzstan, Latvia, Lithuania, Moldova, Russia, Tajikistan, Turkmenistan, Ukraine, and Uzbekistan (former USSR-countries); - papers published from May 1, 2015 to December 31, 2024; - studies describing regions where at least one of the listed above countries was included; - publications in English and Russian languages | - papers not relevant to the defined time frame (from May 1, 2015, to December 31, 2024); - geographically not relevant studies; - publications in local languages apart from English and Russian |
| Intervention | - NAPs on combatting antimicrobial resistance in the examined countries; - country assessments by international ogranisations or country-self assessments | - veterinary or agricultural studies; - laboratory and microbiology research papers; - clinical interventions using antibiotic consumption and antibiotic stewardship; - antiviral, antimycotic, and antifungal consumption; - conference posters and abstracts; - MD (Doctor of Medicine), MPH (Master of Public Health), and PhD (Doctor of Philosophy) dissertations; |
| Comparison | Not applicable | Not applicable |
| Outcomes | - AWaRe classification adoption; - National antimicrobial consumption surveillance; - National antimcirobial stewardship initiatives | - AMR surveillance studies; - Hospital and comminity based studies |
