# Supplementary material for: The Implementation of Antimicrobial Consumption Surveillance and Stewardship in Human Healthcare in Post-Soviet States: A Systematic Review
Source: Antibiotics (Basel). 2025 Jul 25;14(8):749. doi: 10.3390/antibiotics14080749 (PMC12382636; doi:10.3390/antibiotics14080749)
Supplement: Supplementary file 1 [file antibiotics-14-00749-s001.zip › Supplementary material_ Table S1.docx]

**Supplementary file**

**Supplementary file**

Table S1. Included papers

Table S1. Included studies

| **№** | **Authors** | **Title** | **Year** | **Country (-ies)** | **Link, reference** |  |
| --- | --- | --- | --- | --- | --- | --- |
| **Peer-reviewed publications** | | | | | | |
| 1 | Abilova, V., Kurdi, A., & Godman, B. | Ongoing initiatives in Azerbaijan to improve the use of antibiotics; findings and implications. | 2017 | Azerbaijan | https://www.tandfonline.com/doi/full/10.1080/14787210.2018.1417835, [61] |  |
| 2 | Torumkuney D., Kozlov R., Sidorenko S., Kamble R., Lezhnina M., Galushkin A., Kundu S. | Country data on AMR in Russia in the context of community-acquired respiratory tract infections: links between antibiotic susceptibility, local and international antibiotic prescribing guidelines, access to medicine and clinical outcome | 2022 | Russia | https://academic.oup.com/jac/article/77/Supplement_1/i61/6692271, [60] |  |
| 3 | Zhussupova G., Zhaldybayeva S., Skakova A. | Improving the Rational Use of Medicines by Assessing Antibiotic Consumption to Solve the Problem of Antibiotic Resistance in the Republic of Kazakhstan | 2021 | Kazakhstan | https://jhdkz.org/gallery/Special%20issue.%20Number%2040%20(2021).pdf, [62] |  |
| **Grey literature** | | | | | | |
| 4 | Ministry of Healthcare | National Strategy for Combating Antimicrobial Resistance for 2017–2020 | 2024-2026 | Georgia | <https://matsne.gov.ge/en/document/view/6012445?publication=0>, [51] |  |
| 5 | Ministry of Healthcare | Strategy for the Control and Prevention of Antimicrobial Resistance for 2023–2027 | 2023-2027 | Armenia | <https://www.e-draft.am/ru/projects/6011/about>, [50] |  |
| 6 | Ministry of Healthcare | Tajikistan: National Action Plan to Tackle Antimicrobial Resistance in the Republic of Tajikistan. | 2018 | Tajikistan | <https://cdn.who.int/media/docs/default-source/antimicrobial-resistance/who_eng.pdf?sfvrsn=bf5ab498_1&download=true>, [56] |  |
| 7 | Ministry of Healthcare | National Strategy for Containment of Antimicrobial Resistance in Turkmenistan. | 2017 | Turkmenistan | https://www.who.int/publications/m/item/turkmenistan-national-strategy-for-containment-of-antimicrobial- resistance-in-turkmenistan, [57] |  |
| 8 | Ministry of Healthcare | Plan for Combating Antimicrobial Resistance | 2024-2030 | Russian Federation | <https://www.antibiotic.ru/files/337/plan-meropriyatij.pdf>, [55] |  |
| 9 | Ministry of Healthcare | Order on the Approval of the National Program for Surveillance and Control of Antimicrobial Resistance for the Years 2023–2027. | 2023-2027 | Moldova | <https://gov.md/ro/content/hg-proiect-de-hotarare-cu-privire-la-aprobarea-programului-national-pentru-supravegherea-si>, [54] |  |
| 10 | Ministry of Healthcare | One Health Action Plan on Antimicrobial Resistance Containment and Prudent Use of Antibiotics for 2023-2027 | 2023-2027 | Lithuania | <https://likumi.lv/ta/id/343405-antimikrobialas-rezistences-ierobezosanas-un-piesardzigas-antibiotiku-lietosanas-plans-viena-veseliba-2023-2027-gadam>, [24] |  |
| 11 | Ministry of Healthcare | Interagency Programme. Containment of Antimicrobial Resistance in the Kyrgyz Republic for 2022–2025; Food and Agriculture Organization | 2022-2025 | Kyrgyzstan | <https://www.who.int/publications/m/item/kyrgyzstan-national-action-plan-against-antimicrobial-resistance-2022-2025>, [53] |  |
| 12 | Ministry of Healthcare | On Measures to Contain Antimicrobial Resistance in the Republic of Kazakhstan for 2023–2027. | 2023-2027 | Kazakhstan | https://hls.kz/ru/piik-ru, [52] |  |
| 13 | Ministry of Healthcare | One Health Action Plan on Antimicrobial Resistance Containment and Prudent Use of Antibiotics for 2023-2027 | 2023-2027 | Latvia | https://likumi.lv/ta/id/343405-antimikrobialas-rezistences-ierobezosanas-un-piesardzigas-antibiotiku-lietosanas-plans-viena-veseliba-2023-2027-gadam, [23] |  |
| 14 | Ministry of Healthcare | National Action Plan to Combat Antimicrobial Resistance in Ukraine, Approved by Cabinet Resolution No.1614 | 2024 | Ukraine | <https://moz.gov.ua/uk/vsesvitnij-tizhden-obiznanosti-pro-stijkist-do-protimikrobnih-preparativ-yak-moz-posilyuye-nacionalnu-politiku-borotbi-z-antimikrobnoyu-rezistentnistyu>, [58] |  |
| 15 | World Health Organisation | AMC Network | 2023 | WHO European region countries | https://www.who.int/europe/publications/i/item/9789289061346 [63] |  |
| 16 | World Health Organisation | AMC Network | 2024 | WHO European region countries | https://www.who.int/europe/publications/i/item/WHO-EURO-2024-10997-50769-76929, [17] |  |
| 17 | World Health Organisation | GLASS | 2022 |  | https://worldhealthorg.shinyapps.io/glass-dashboard/_w_05cb994ae21f4b06a6e69844bc3665e6/#!/amu, [20] |  |
| 18 | European Center of Disease Control | ESAC-Net. European Surveillance of Antimicrobial Consumption Network |  | 25 European Union Member States and two European Economic Area countries (Iceland and Norway) | https://www.ecdc.europa.eu/en/about-us/partnerships-and-networks/disease-and-laboratory-networks/esac-net, [18] |  |
| 19 | World Health Organisation, United Nations Environment Programme, Food and Agriculture Organization, and World Organisation for Animal Health | Tracking antimicrobial resistance country self-assessment survey | 2023 | All countries | https://amrcountryprogress.or g/#/map-view, [12] |  |
| 20 | World Health Organisation | Review of Antibiotics in National Medicines Selection Lists in Eastern Europe and Central Asia. | 2023 | All countries | https://iris.who.int/bitstream/handle/10665/365989/9789289058582-eng.pdf, [21] |  |
| 21 | World Health Organisation | Joint external evaluation of IHR core capacities of Azerbaijan | 2023 | Azerbaijan | https://iris.who.int/bitstream/handle/10665/375902/9789240082434-eng.pdf?sequence=1&isAllowed=y, [13] |  |
| 22 | World Health Organisation | Joint external evaluation of IHR core capacities of Georgia | 2024 | Georgia | https://iris.who.int/bitstream/handle/10665/380216/9789240102941-eng.pdf?sequence=1&isAllowed=y, [14] |  |
| 23 | World Health Organisation | Joint external evaluation of IHR core capacities of Kyrgyzstan | 2023 | Kyrgyzstan | https://iris.who.int/bitstream/handle/10665/376260/9789240090286-eng.pdf?sequence=1&isAllowed=y, [15] |  |
| 24 | World Health Organisation | Joint external evaluation of IHR core capacities of Armenia | 2023 | Armenia | https://iris.who.int/bitstream/handle/10665/376959/9789240093843-eng.pdf?sequence=1&isAllowed=y, [19] |  |
| 25 | World Health Organisation | Joint external evaluation of IHR core capacities of Estonia | 2023 | Estonia | https://iris.who.int/bitstream/handle/10665/376723/9789240092921-eng.pdf?sequence=1&isAllowed=y, [45] |  |
| 26 | World Health Organisation | Joint external evaluation of IHR core capacities of Uzbekistan | 2022 | Uzbekistan | https://iris.who.int/bitstream/handle/10665/367173/9789240070165-eng.pdf?sequence=1&isAllowed=y, [59] |  |
| 27 | World Health Organisation | Joint external evaluation of IHR core capacities of Tajikistan | 2019 | Tajikistan | https://iris.who.int/bitstream/handle/10665/339323/9789240018242-eng.pdf?sequence=1&isAllowed=y, [16] |  |
| 28 | World Health Organisation | Joint external evaluation of IHR core capacities of Lithuania | 2018 | Lithuania | https://iris.who.int/bitstream/handle/10665/311201/WHO-WHE-CPI-2019.35-eng.pdf?sequence=1&isAllowed=y [46] |  |
| 29 | World Health Organisation | Joint external evaluation of IHR core capacities of Moldova | 2018 | Moldova | https://iris.who.int/bitstream/handle/10665/311711/WHO-WHE-CPI-2019.54-eng.pdf?sequence=1&isAllowed=y, [47] |  |
| 30 | World Health Organisation | Joint external evaluation of IHR core capacities of Turkmenistan | 2016 | Turkmenistan | https://iris.who.int/bitstream/handle/10665/255632/WHO-WHE-CPI-2017.29-eng.pdf?sequence=1&isAllowed=y, [48] |  |
| 31 | World Health Organisation | Joint external evaluation of IHR core capacities of Latvia | 2017 | Latvia | https://iris.who.int/bitstream/handle/10665/258969/WHO-WHE-CPI-2017.27.report-eng.pdf?sequence=1&isAllowed=y, [49] |  |
